# Fluorescently Labeling Amino Acids in a Deep Eutectic Solvent

**DOI:** 10.1021/acs.analchem.2c03980

**Published:** 2022-11-22

**Authors:** Jessica Torres, Karen S. Campos, Christopher R. Harrison

**Affiliations:** Department of Chemistry and Biochemistry, San Diego State University, 5500 Campanile Drive, San Diego, California 92182, United States

## Abstract

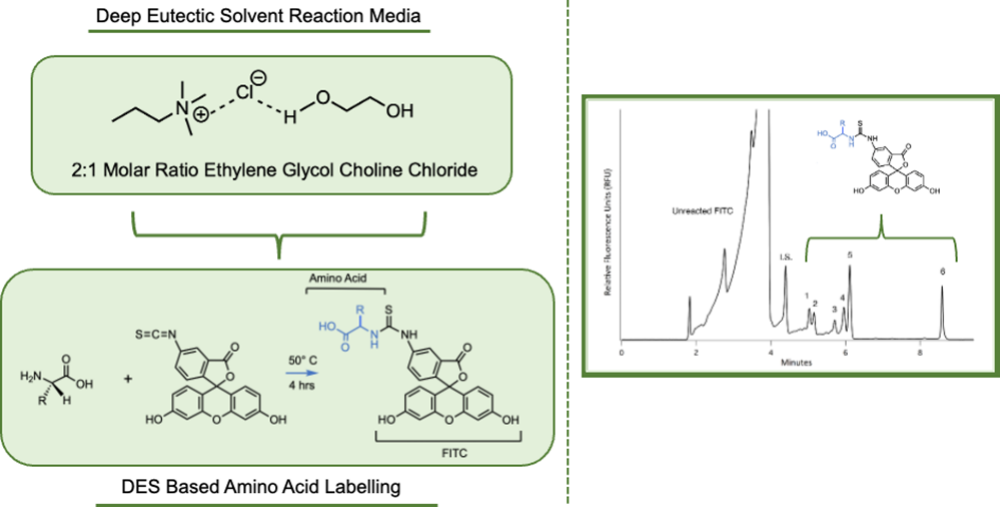

The increased use of deep eutectic solvents (DESs) in recent years has been significant and provides new approaches to sample collection and preparation. At the same time, the use of these new solvents to prepare samples can present challenges for subsequent analyses. Common analytical approaches, such as fluorescent labeling, may not be compatible with the solvents. In this work, we explore how effective three traditional fluorescent labels can be at derivatizing amino acids in the most common DESs, formed from choline chloride and ethylene glycol. We demonstrate that the unique solvent characteristics of the DESs still allow for two of the fluorophores, fluorescein isothiocyanate and 5-carboxyfluorescein succinimidyl ester, to effectively label amino acids. Initial optimizations of the reaction conditions demonstrate that we can effectively label both d- and l-amino acids, in solution with concentrations of amino acids down to 4 μM. Capillary electrophoretic separations following this preparation can detect as little as 50 nM. This is possible without removal of any DES from the sample matrix. These results represent the first complete fluorescent labeling reaction in a DES and subsequent capillary electrophoretic separation of the analytes.

The use of deep eutectic solvents (DESs) has grown significantly in a broad range of applications from green solvents to improved chromatographic separations. DESs are an incredibly broad range of solutions prepared from specific molar ratios of a hydrogen bond donor (e.g., choline chloride,^1^ glucose,^2^ betanine^3^) and hydrogen bond acceptor (e.g., urea,^3^ ethylene glycol,^4^ citric acid^2^). It has been speculated that there are thousands of possible combinations of unique DESs; however, the majority of the published work uses the combination of ethylene glycol (EG) and choline chloride (ChCl), termed ethaline.^5^ The defining factor of all deep eutectic solvents, aside from the binary composition of a hydrogen bond donor and acceptor, is their lowered melting point. In many instances, the DES is prepared from solids, which when combined will become liquid at room temperature. Though seemingly trivial to prepare, there are challenges and subtleties to the preparations of some of these mixtures, particularly when heating is required to initiate the self-dissolution of the solids.^6^ A potential complication with choline chloride based natural deep eutectic solvents is that they may start to decompose at high temperatures rendering the DES ineffective.^7^

Our interest in the use of DESs arises from their low melting points and low vapor pressures. As we develop techniques to explore the surface of other planetary bodies for chemical traces of past life, a solvent which can remain liquid at low temperatures and pressure could be invaluable. We would like to be able to extrude a DES from a rover into pores and cracks in Martian rocks to be able to explore these spaces for chemical traces of past life. Should the DES be able to solubilize likely chemical signatures of life, such as amino acids, and then allow them to be fluorescently labeled, we would be able to explore new environments in the search for past life.

Therefore, we are exploring how effectively common fluorescent probes can label amino acids when both the probe and the amino acid have been dissolved in the ethaline DES (2:1 mol ratio of EG and ChCl). Our tests include three common fluorophores: fluorescein isothiocyanate (FITC),^8−11^ 5-carboxyfluorescein succinimidyl ester (CFSE),^12−16^ and 4-chloro-7-nitrobenzofurazan (NBD-Cl).^17,18^ These have been selected both due to their routine use, as well as for the variations in the typical reaction conditions employed for each. FITC reactions are typically carried out by dissolving the dye in anhydrous DMSO or acetone and adding it to the target amines in an aqueous carbonate buffered (pH 8.3) solution.^11,19^ Similarly, CSFE labeling reactions are carried out by dissolving the reactive dye in DMSO or DMF, while the target analytes are dissolved in an aqueous, amine free buffer between pH 7–9.^12−16^ NBD-Cl is most effective at labeling amino acids when the dye is dissolved in methanol, and the target is dissolved in an aqueous borate buffer at pH 9–10.^20,21^ As targets for these labeling reactions, we have selected a range of d- and l-amino acids. In addition, we have explored how the adjustment of the pH of the DES and the reaction temperature impact the labeling reactions performed in ethaline DES.

## Results and Discussion

To evaluate the viability of the commonly used amine reactive fluorophores, FITC, NBD-Cl, and CFSE, the first test was performed by directly combining the dyes with serine (Ser) in a pure ethaline solvent. In the reaction vial, which contained 100 μL of total solution, the Ser concentration was 100 μM, and a 4:1 excess of each dye was used in the respective tests. The solutions were allowed to react for 4 h at room temperature. Once the reaction period was complete, 50 μL of the solution was taken and mixed with 1 μL of 1 mM fluorescein as an internal standard and 149 μL of DI water to dilute the solution and reduce the viscosity for injection. This dilution with water and the amount of the internal standard were kept constant for all subsequent analyses. The dilution of the ethaline reaction mixture was done to reduce the viscosity of the DES solution, to both facilitate the hydrodynamic injection of the samples and to minimize the band broadening that occurs with significant viscosity difference between the sample zone and BGE.^22^

**Figure 1:**
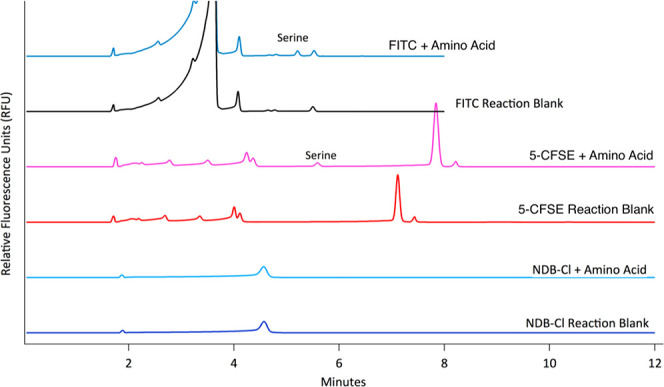
Representative electropherograms of 100 μM l-Ser labeled with NDB-Cl, 5-CFSE, and FITC with respective dye blanks (The injected concentration of l-Ser is 25 μM.). Separations were done with 40 cm, 50 μm i.d. capillary at +25 kV. BGE used for separation was 50 mM sodium tetraborate pH 9.4. The internal standard was 5 μM fluorescein (4 min).

Figure 1 is a collection of representative electropherograms obtained for each of the three dyes when the reactions were performed in the presence and absence of Ser. It is clearly evident that the NDB-Cl is not compatible with these reaction conditions, as no changes are seen between the blank and Ser containing reactions. Given the successes that we had with both FITC and CFSE, we opted to focus on those reagents as the most promising ones for our purposes.

Though both FITC and CFSE are able to directly react with Ser in the pure ethaline solvent and yield similar intensities for the labeled amino acid, the FITC reagent was selected as the reagent for further optimization and investigation. This selection was made due to the fact that we experienced difficulties in obtaining consistent and reproducible results with the CFSE dye. There appears to be an issue with the stability of CFSE in ethaline solutions, as the reaction became less consistent with the age of the solution, a problem that was not experienced with the FITC dye.

Though the FITC dye was capable of labeling amino acids in a pure DES solvent, the efficiency of this labeling was far less than what was seen with the same amino acid and dye prepared in conventional solvents. One factor to consider in the derivatization reaction is the deprotonation of the primary amine. Traditional labeling reaction conditions with FITC are carried out with a sodium carbonate buffer at pH 8.3 or sodium tetraborate at pH 9.4 to facilitate this process.^9−11^ Marcus has stated that one chemical solvent property of deep eutectic solvents is their inherent acidity, which renders the deprotonation of acids less effective.^23^ This may be complicating the labeling of the amino acids, as ethaline should be far more acidic than traditional reaction solvents.

As there are no calibration standards for pH meter measurements of DESs, the pH of a DES cannot be measured as accurately as that of an aqueous solvent. For this reason, DESs are given an apparent pH, an approximation of their pH.^23^ We opted to use universal pH indicator strips to measure the apparent pH of the DES, as this allows for comparisons when the solvent was modified. The apparent pH of pure ethaline when measured this way is about 5, though given the successful labeling it is likely that the effective pH is actually higher. To further improve the yield of amino acid labeling, we aim to increase the pH into the range of 8–11, to better represent the pH of traditional labeling methods. To do this, several buffers and bases were selected for the initial pH adjustment of ethaline. We initially kept the total concentration of the pH additives constant at 10 mM for consistency. Each additive was used as its solid and dissolved in pure ethaline. The amount of solid to add was determined based on targeted pH and the known aqueous dissociation constants for each base or buffer. Table 1 lists the tested additives and the apparent pH that was measured for each solution; however, none of the mixtures that were prepared reached their targeted pH. The closest was the carbonate buffer, with an apparent pH 1.3 units below the target; the furthest was the phosphate buffer reaching a pH 4.3 units below the target value. Similarly, solutions of KOH failed to achieve the desired pH when prepared in ethaline.

**Table 1:** Influence of pH Additive on FITC Labeling of Amino Acids in DESs

reaction solvent solution	calculated pH	apparent pH^a^	l-Ser peak area^b^ [*n* ≥ 4]	l-Leu peak area^b^ [*n* ≥ 4]
pure ethaline		5	1.00 ± 0.05	1.00 ± 0.05
10 mM NaHCO_3_/Na_2_CO_3_	10.3	9	0.20 ± 0.04	
10 mM Na_2_HPO_4_/Na_3_PO_4_	12.3	8	1.1 ± 0.3	
10 mM B_4_Na_2_O_7_	9.4	6	2.05 ± 0.04	
10 mM KOH	12.0	9	2.3 ± 0.3	3.6 ± 0.1
5 mM KOH	11.7	8	7.8 ± 0.1	9.3 ± 0.1
1 mM KOH	11.0	6	2.3 ± 0.1	1.58 ± 0.01

a Apparent pH measurements taken via universal indicator pH strips.

b Peak areas are normalized to that obtained in pure ethaline and measured as the ratio of the peak area relative to the internal standard.

**Figure 2:**
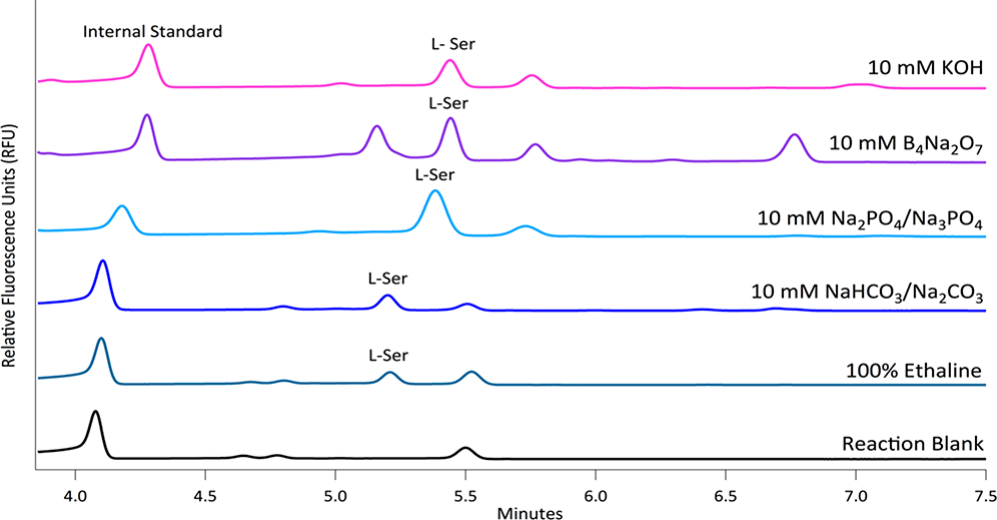
Representative electropherograms of 100 μM l-Ser reacted with FITC in different 10 mM base/buffer adjusted ethaline solutions, a pure ethaline solution, and a reaction blank (The injected concentration of l-Ser is 25 μM.). Separations were done with 40 cm, 50 μM i.d. capillary at +25 kV. BGE used was 50 mM sodium tetraborate pH 9.4. The internal standard (IS) was 5 μM fluorescein.

However, there was a clear improvement in the labeling efficiency for all pH modified ethaline reaction conditions (Figure 2). The quantification of the improvement in the labeling can be seen in Table 1, for the reactions with both Ser and leucine (Leu). From this comparison, it is evident that 10 mM KOH solution yields the greatest improvement in the reaction efficiency, with an apparent pH of 9, and a doubling of the labeling efficiency as compared to pure ethaline. Though sodium tetraborate showed comparable labeling efficiencies, we opted to use KOH for further pH optimization.

**Figure 3:**
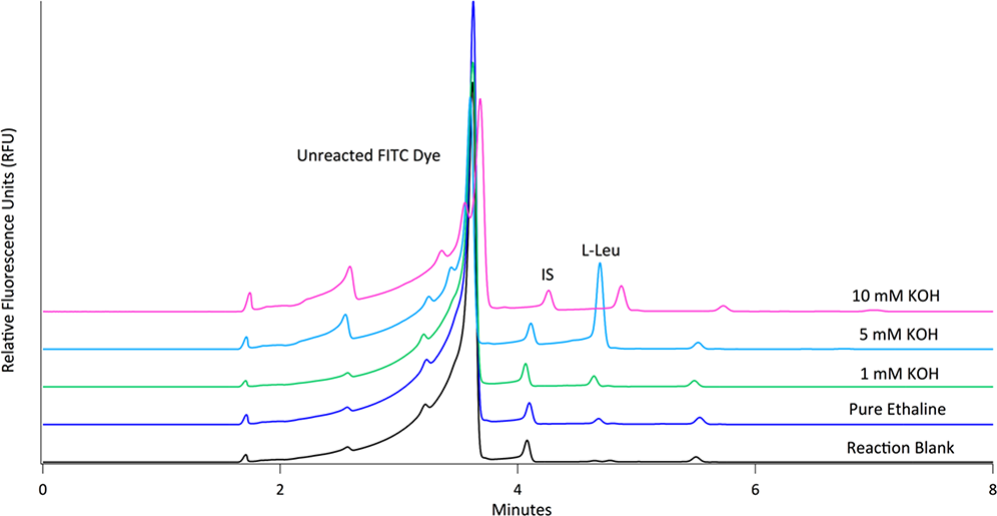
Representative electropherograms of 100 μM l-Leu (4.8 min) reacted with FITC in 1, 5, and 10 mM KOH adjusted ethaline DES, along with a reaction blank (The injected concentration of l-Leu is 25 μM.). Separations were done with 40 cm, 50 μm i.d. capillary at +25 kV. BGE used was 50 mM sodium tetraborate pH 9.4. The internal standard (IS) was 5 μM fluorescein (∼4 min).

As the addition of KOH improved the labeling reaction yield, further experiments were undertaken to determine the optimal concentration of KOH to add to the reaction. A range of 1–100 mM KOH was selected for this process. Previous work has shown that in higher pH solutions there is an increased likelihood of FITC reacting to form a hydrolysis product and being less viable for the labeling reaction of amino acids.^12,13^ The ethaline solution with 100 mM KOH produced an apparent pH of at least 10, and this appears to have resulted in the formation of the anticipated hydrolysis products of the FITC dye as there was an increase in the amount of dye byproducts and no evidence of labeling, making it unfit for labeling reactions. The optimization thus focused on the lower concentrations of KOH. The apparent pH’s of 1, 5, and 10 mM KOH in ethaline were measured as 6, 8, and 9, respectively; these solutions were used for labeling l-Leu as well as l-Ser (Table 1). Figure 3 shows representative electropherograms of the products of 100 μM l-Leu being reacted in ethaline with either 1, 5, or 10 mM KOH. From these results, it is clear that a 5 mM concentration of KOH is optimal for the reaction of FITC with amino acids. Compared to the reaction in pure ethaline, 5 mM KOH yielded a 7.8-fold increase in the l-Ser peak area and a 9.3-fold increase in the l-Leu peak area (Table 1). The trend in our data would indicate that the formation of the aforementioned FITC hydrolysis products begins somewhere between 5 and 10 mM KOH. Thus, we opted to use 5 mM KOH for the remainder of our analyses.

**Figure 4:**
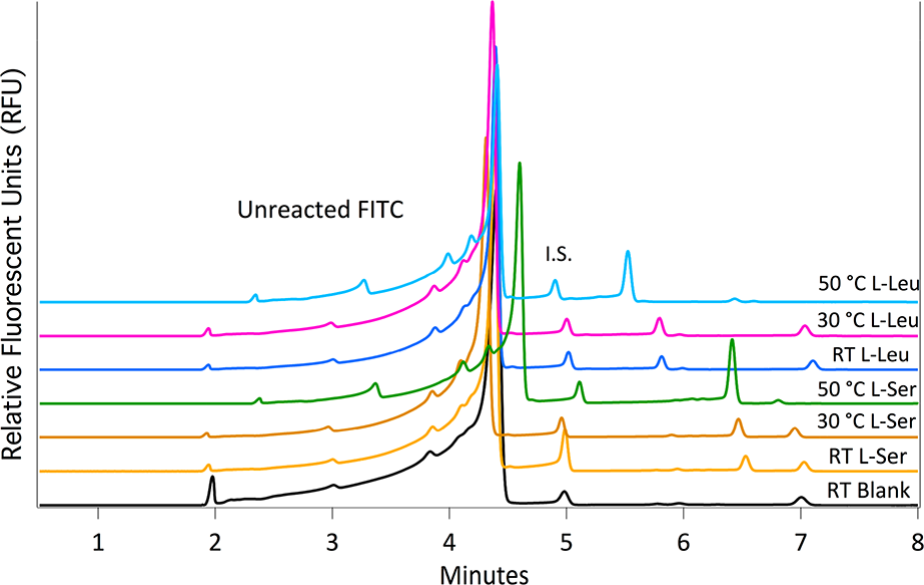
Representative electropherograms of 100 μM l-Leu (5.5–5.8 min) and l-Ser (6.4–6.6 min) in 5 mM KOH ethaline reacted with FITC at RT, 30 °C, and 50 °C, along with a reaction blank (The injected concentration of amino acid is done at 25 μM.). Separations were done with 40 cm, 50 μm i.d. capillary at +25 kV. BGE used was 50 mM sodium tetraborate pH 9.4. The internal standard was 5 μM fluorescein (∼5 min).

Finally, we investigated how increases in the reaction temperature might further increase the yield of the reaction. For these tests, the 5 mM KOH in ethaline solution was used, and the reaction was performed at RT, 30 °C, and 50 °C. Higher temperatures were not investigated as it has been shown that at temperatures greater than 60 °C there is degradation of choline chloride.^7^Table 2 shows a comparison of l-Ser and l-Leu reactions at a range of temperatures, and it is clear that heating the solution has a positive impact. The peak areas for l-Ser and l-Leu increase by factors of 8 and 4, respectively, when comparing the RT reaction to that at 50 °C. An added benefit of the heating is an overall reduction in the variance of the reaction; the modest fluctuations in RT resulted in an RSD of 80% for the l-Ser labeling process. At present, it is unclear why the Ser is more effectively labeled at elevated temperatures, though we suspected the size and/or polarity of the side chain play a role. Representative electropherograms for the reactions of 100 μM concentrations of the amino acids at the varying temperatures can be seen in Figure 4. Along with the increase in the labeled amino acid peak areas, we also observed a decrease in the FITC peak at ∼7 min. All subsequent labeling reactions are to be carried out at 50 °C to ensure optimal conversion to the amino acid-FITC complex.

**Table 2:** Influence of Temperature on the FITC Labeling of Amino Acids in Ethaline with 5 mM KOH^a^

reaction temp	l-Ser normalized peak area [*n* ≥ 6]	l-Leu normalized peak area [*n* ≥ 6]
RT	0.5 ± 0.4	0.6 ± 0.1
30 °C	1.1 ± 0.4	0.9 ± 0.3
50 °C	4.2 ± 0.8	2.6 ± 0.1

a The amino acid peak area is normalized relative to that of the fluorescein internal standard, which is a constant 5 μM concentration.

As the increase of temperature and the pH adjustment of the ethaline-based reaction improved the labeling of our test amino acids, a larger group of nonpolar, polar, and uncharged amino acids was selected to test the limitations of this labeling process. Additionally, both the d- and l-enantiomers were tested, as we would ultimately like to apply this procedure to the analysis of the enantiomeric composition of amino acid mixtures. The results of the labeling reactions are presented in Table 3, where the normalized peak area for each enantiomeric pair is compared. Of the five enantiomeric amino acids tested only serine and histidine yielded statistically equivalent peak areas. With alanine, leucine, and glutamic acid, the d-enantiomer yielded a greater signal following the labeling reaction. At present, we have not been able to identify a reason for the difference in the labeling efficiencies of the enantiomers.

**Table 3:** Comparison of FITC Labeling of Chiral Amino Acids in Ethaline

amino acid	d-peak area^b^ [*n* ≥ 6]	l-peak area^b^ [*n* ≥ 3]	*t* test
alanine	6.9 ± 0.2	3.9 ± 0.1	not equivalent
glutamic acid	7.65 ± 0.09	7.1 ± 0.2	not equivalent
glycine^a^	1.21 ± 0.03		
histidine	4.3 ± 0.5	4.0 ± 0.1	equivalent
leucine	4.3 ± 0.1	3.5 ± 0.4	not equivalent
serine	10 ± 2	9 ± 1	not equivalent

a Glycine is achiral.

b The amino acid peak area is normalized relative to that of the fluorescein internal standard, which is a constant 5 μM concentration.

**Figure 5:**
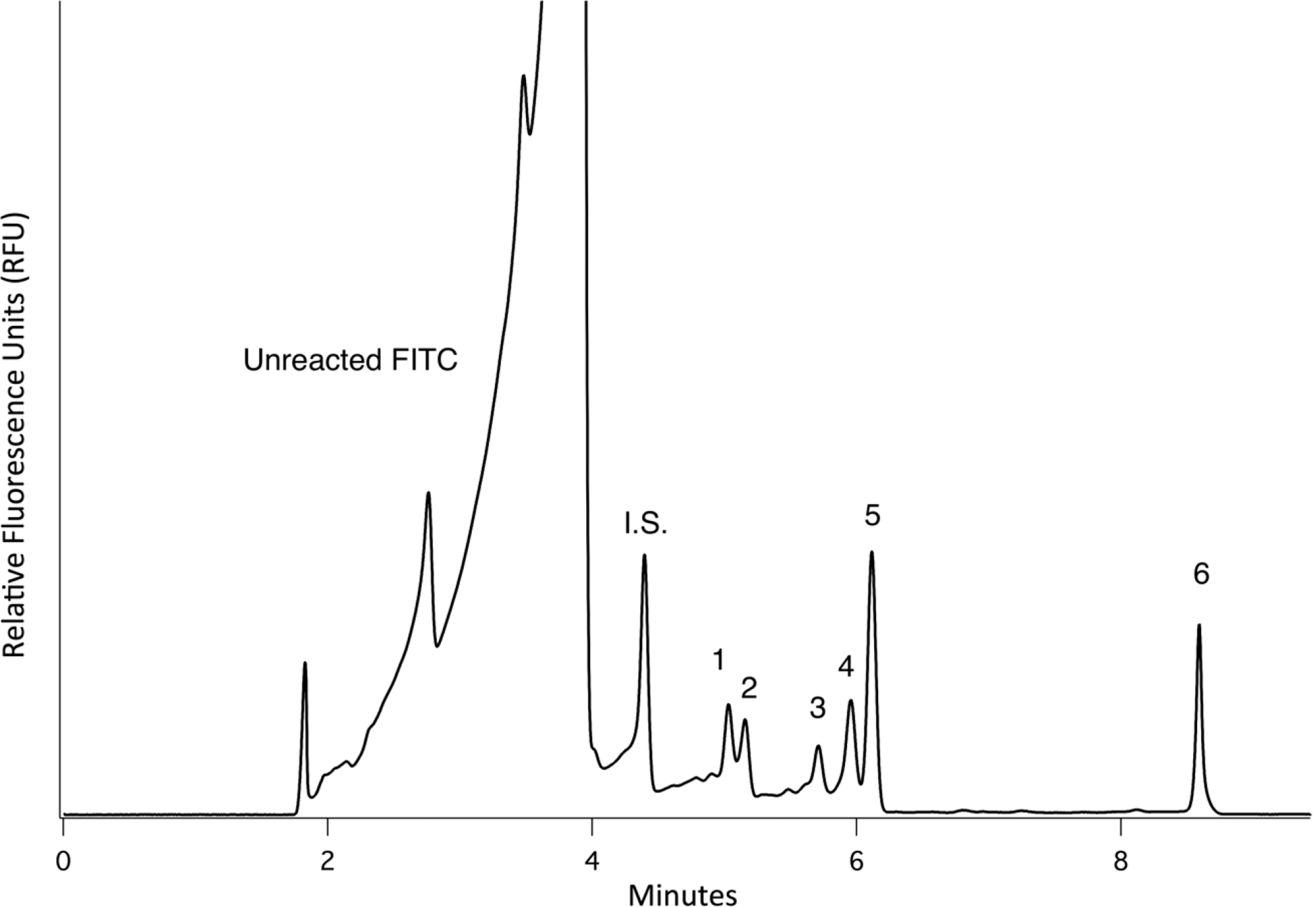
Representative electropherograms of various amino acids simultaneously reacted in 5 mM KOH ethaline at (1) Leu, (2) l-His, (3) l-Ser, (4) l-Ala, (5) Gly, and (6) l-Glu, all at 25 μM amino acid with FITC (The injected concentration of each individual amino acid is 6.3 μM.). Under these conditions, there is coelution between the (5) Gly peak and unreacted FITC peak. Separations were done with 40 cm, 50 μm i.d. capillary at +25 kV. BGE used was 50 mM sodium tetraborate pH 9.4. The internal standard (I.S.) was 5 μM fluorescein.

To demonstrate that the labeling in modified ethaline is possible with mixed amino acid samples, Figure 5 shows a representative electropherogram of a mixed sample of l-Ala, l-Glu, Gly, l-His, l-Leu, and l-Ser at 6.3 μM. All amino acids were mixed to a concentration of 6.3 μM prior to undergoing the labeling reaction with our optimized conditions. Though the separation conditions have not been optimized, there is clear separation and near baseline resolution for all of the FITC labeled amino acids. The migration order of the FITC labeled amino acids also matches that shown by others with similar BGE conditions,^9−11^ demonstrating that the ethaline sample matrix does not drastically influence the separation. While chiral separation is not feasible with this specific buffer, it is anticipated that the addition of chiral selectors will allow for their separation in the future studies.

## Conclusion

This work shows that it is possible for ethaline DES to act as a solvent for the fluorescent labeling reaction of nonpolar, polar, and uncharged d/l-amino acids. This data shows the example of performing fluorescent labeling of amino acids in a deep eutectic solvent followed by their direct separation from this matrix via capillary electrophoresis. With this labeling method, we have been able to derivatize amino acids at concentrations as low as 4 μM. We have also been able to perform CE-LIF detection of as little as 50 nM labeled amino acids in a DES-based sample matrix. While our optimization of this reaction has been effective, there is significant work still to be done to explore how DESs can be used in analytical chemistry and capillary electrophoresis. Our work has thus far only focused on the most common DES, ethaline, yet there are multitudes of other DESs that exist and may yield even greater benefits to a wide range of applications. We hope that our work provides a framework and inspiration to others to explore the further use of DESs for labeling reactions and separations.

## Supporting Information Available

Experimental conditions and instruments (PDF)
