# Supplementary material for: Fluorescently Labeling Amino Acids in a Deep Eutectic Solvent
Source: Anal Chem. 2022 Nov 22;94(48):16538–42. doi: 10.1021/acs.analchem.2c03980 (PMC9730294; doi:10.1021/acs.analchem.2c03980)
Supplement: Supplementary file 1 — ac2c03980_si_001.pdf [file ac2c03980_si_001.pdf]

Supporting Information

Fluorescently labelling amino acids in a deep eutectic solvent.

Jessica Torres, Karen S. Campos, Christopher R. Harrison*

AUTHOR ADDRESS

Department of Chemistry and Biochemistry, San Diego State University, 5500 Campanile Drive, San Diego, California, 92182, United States.

ABSTRACT: This supporting information contains the experimental details for this work. Including the list and source of the reagents and materials used, the instrumentation employed, and the procedures for the labelling of the amino acids in the deep eutectic solvent.

Experimental Section

Reagents. Extra pure ethylene glycol, 99+%, pure fluorescein isothiocyanate isomer I, 90% L- and D- leucine, L-, and D- alanine, L- histidine, L- and D- valine, L- and D- serine, L-(+)- glutamic acid, and glycine were purchased from Acros Organics (Fair Lawn, NJ, USA). D- histidine was purchased from TCI Chemicals (Tokyo, Japan). D- glutamic acid, and choline chloride were purchased from Alfa Aesar (Haverhill, MA, USA). Fluorescein was purchased from EMD Chemicals Inc. (Darmstadt, Germany). Buffer preparation was done with sodium tetraborate decahydrate, 99+% purchased from Alfa Aesar (Ward Hill, MA, USA). Sodium hydroxide pellets were purchased from EMD Millipore (Darmstadt, Germany). HPLC grade methanol and hydrochloric acid was purchased from Fisher Scientific (Fair Lawn, NJ, USA). 5-carboxyfluorescein (CFSE) was purchased from Invitrogen (Eugene, OR, USA). NBD chloride, 98%, fluorescein isothiocyante isomer I, 90% pure, and sodium tetraborate, 98% anhydrous were purchased from Acros Organics (Fair Lawn, NJ, USA).

Apparent pH was measured using Fisher brand plastic pH indicator strips for 0.0-14.0, pH strips were purchased from Fisher Scientific (Fair Lawn, NJ, USA). Adjustments made to the ethaline were done with ACS grade sodium carbonate, sodium carbonate anhydrous, sodium phosphate dibasic anhydrous, sodium phosphate tribasic dodecahydrate purchased from EMD Millipore (Darmstadt, Germany). Sodium tetraborate 98% anhydrous was also used for pH adjustment purchased from Acros Organics (Fair Lawn, NJ, USA). Reagent grade potassium hydroxide pellets for pH adjusted ethaline was purchased from Fisher Scientific (Rochester, NY, USA).

Instrumentation. Separations were performed on a Beckman Coulter ProteomeLab PA 800 with LIF detection (488 nm) (Brea, CA). 32-Karat software was used. Fused silica capillaries (40 cm × 50 μm i.d. × 360 μm o.d.) were purchased from Polymicro Technologies (Phoenix, AZ, USA). The capillary was prepared by burning a small window opening into the polyimide coating 10 cm from the capillary outlet for detection. The capillary was conditioned by rinsing for 10 min each with methanol, water, 3 M HCl, water, 3 M NaOH, water, and the background electrolyte (BGE). Pressure injections of 0.5 psi for 12.0 s were used. Voltage separation used was 25 kV. All solutions were prepared using 18.2 MW water from the Milli-Q Academic water filtration system (Millipore). Adjustments made to a buffer's pH were quantified using the Fischer Scientific Accumet® basic AB15 pH meter (Pittsburgh, PA, USA).

Derivatization of amino acids. The preparation of the deep eutectic solvent, a 2:1 molar ratio mixture of ethylene glycol and choline chloride (ethaline), was accomplished by adding 30.0 mL of ethylene glycol to 38.87 g of choline chloride in a beaker. The two components were mixed using a stir-bar at 450 rpm at room temperature until homogenized. Separate pH-modified ethaline mixtures were prepared by dissolving appropriate amounts of solid KOH, $B_4Na_2O_7$, Na_2PO_4/Na_3PO_4, or $NaHCO_3$/Na_2CO_3 directly into the ethaline. The direct measurement of the ethaline solution pH, with a digital pH meter, was not deemed viable as appropriate calibration solutions, prepared in the same DES, are not available. The calculations performed to determine the appropriate amount and ratios of the additives needed to obtain the desired pH were all based on known aqueous dissociation constants for all components. These dissociation constants are unlikely to be the same in ethaline, however this was deemed the best approach for consistent modifications of the DES pH.

Stock 1 mM solutions of D- or L- amino acids were prepared by weighing the required amounts of the amino acids and dissolving the solid in 10.00 mL of ethaline, delivered via a 10 mL Eppendorf pipette. To ensure the complete dissolution of the amino acids the mixtures were sonicated with a Fisher Scientific FS20 Ultrasonic Cleaner (Waltham, MA, USA) for 15-20 minutes. Similarly, when needed a 1 mM stock solution of fluorescent dye was prepared by dissolving the necessary amount of solid dye in 5-10 mL of ethaline dispensed via pipette, and sonicating the solution for 5 minutes. The fluorescent dye solutions were stored in the freezer when not in use and allowed to defrost to room temperature prior to use. Amino acid stocks were stored in glass vials at room temperature in the dark.

For the labelling reactions of individual amino acids with FITC, the reactions were, unless otherwise indicated, carried out in amber vials by combining 10 μL of the desired 1 mM amino acid and 40 μL of 1 mM FITC dye along with 50 μL of either pure, or pH-adjusted, ethaline. The solutions were allowed to react for 4 hours at set temperatures between 25 and 50 ˚C. Unused samples were stored in the fridge until time for analysis. For the CE-LIF analysis of the reaction products, 50 μL of the labelled amino acid mixture was mixed with 1 μL of 1 mM fluorescein (internal standard), and 149 μL of water, in a 250 μL sample vial.
